# Semi-Automatic System for ZnO Nanoflakes Synthesis via Electrodeposition Using Bioinspired Neuro-Fuzzy Control

**DOI:** 10.3390/biomimetics10100712

**Published:** 2025-10-21

**Authors:** Yazmín Mariela Hernández-Rodríguez, Yunia Veronica Garcia-Tejeda, Esperanza Baños-López, Oscar Eduardo Cigarroa-Mayorga

**Affiliations:** 1 Department Advanced Technologies, UPIITA-Instituto Politécnico Nacional, Av. IPN 2580, CDMX, Ciudad de Mexico C.P. 07340, Mexico; yazmin.hernandez@cinvestav.mx (Y.M.H.-R.); ygarciat@ipn.mx (Y.V.G.-T.); 2 Academia de Química, Universidad Autónoma del Estado de Hidalgo (UAEH), Carretera Pachu-ca-Tulancingo Km. 4.5., Hidalgo C.P. 42184, Mexico; esperanza_banos10303@uaeh.edu.mx

**Keywords:** electrodeposition, ZnO nanostructures, neuro-fuzzy control (ANFIS), electrochemical synthesis, microflake morphology

## Abstract

This research presents the development and characterization of a semi-automatic electrophoretic deposition (EPD) system designed for the synthesis of zinc oxide (ZnO) microstructures, utilizing a bioinspired neuro-fuzzy control strategy (ANFIS). The system was designed based on a chemical reactor regulated by electricity in a potentiostate cell to automate and optimize the deposition parameters by controlling the temperature. The synthesized ZnO coatings exhibited distinctive flake-like morphology, confirmed via Scanning Electron Microscopy (SEM), X-Ray Diffraction (XRD), and Energy-Dispersive X-Ray Spectroscopy (EDS), validating their morphological uniformity and compositional consistency. The implemented ANFIS controller was trained using experimentally acquired data, making a correlation with the properties of the sample, thickness and porosity, also employed as inputs of the system. The system exhibited high accuracy in predicting optimal deposition conditions for ZnO nanoflakes obtention, specifically in the temperature-dependent variations in thickness and porosity employed as reference to establish four classes of working sets based on the density of ZnO flakes in the substrate. Results indicate that the bioinspired neuro-fuzzy control substantially enhances the adaptability and predictive capabilities of the electrophoretic deposition process, making it a versatile tool suitable for various applications requiring precise microstructural characteristics. Future directions include further refinement of the control system, incorporation of digital sensing technologies, and potential expansion of the platform to accommodate other functional materials and complex deposition scenarios.

## 1. Introduction

The synthesis of micro and nanostructured materials has gained immense relevance in recent years, driven by their unique physical, chemical, and mechanical properties, which differ significantly from their bulk counterparts [1,2]. These properties have opened unprecedented avenues for applications across diverse fields such as electronics, medicine, environmental remediation, energy storage, and catalysis [3,4,5]. The effectiveness of micro and nanostructures in these applications is often critically dependent on precise control over morphology, size, distribution, and crystallinity, thus necessitating the development of advanced and reliable synthesis methodologies [6]. Traditionally, synthesizing micro and nanostructured materials involves manual or semi-manual processes, where human intervention is frequently required to monitor and adjust experimental parameters [7]. While effective at the laboratory scale, these manual approaches suffer from reproducibility issues, limited scalability, and variability in product quality [8]. Moreover, the precision required for obtaining consistently desirable properties can be challenging to achieve manually due to the sensitivity of synthesis processes to slight variations in parameters such as temperature, concentration, pH, electric fields, and deposition time [9]. To address these challenges, there has been growing interest in integrating automation into the synthesis procedures. Automatic and semi-automatic synthesis systems offer substantial advantages, including enhanced reproducibility, improved quality control, reduced human error, and increased scalability from laboratory research to industrial production [10]. Additionally, automation facilitates systematic exploration of synthesis conditions through high-throughput experimentation, significantly accelerating material discovery and optimization processes [11]. Among the various methods available, electrochemical techniques, particularly electrophoretic deposition (EPD), have emerged as attractive options for the controlled fabrication of micro and nanostructured materials [12]. Electrophoretic deposition is a versatile and relatively simple technique in which charged particles suspended in a liquid medium migrate under an applied electric field toward an electrode, forming a dense and uniform coating. EPD provides excellent control over film thickness, uniformity, and microstructure, making it particularly suitable for fabricating complex geometries and functional coatings for sensors, biomedical implants, corrosion-resistant surfaces, and energy storage devices [12,13]. In the first step of typical ZnO EPD, hydroxyl ions are generated at the cathode as a result of the reduction of dissolved oxygen or nitrate ions, depending on the electrolyte composition. In our system, the predominant process is the reduction of dissolved oxygen under the applied potential. In the second step, these hydroxyl ions react with zinc ions in the solution to form zinc hydroxide, which subsequently dehydrates under the deposition temperature to yield zinc oxide. This process is strongly influenced by factors such as the applied potential, electrolyte pH, and temperature, which govern the nucleation rate, growth kinetics, and the resulting morphology of ZnO, leading to structures such as flakes, rods, or other nanostructures. By briefly incorporating this mechanism, the introduction now provides a clearer scientific foundation for understanding the role of the semi-automatic control system and the ANFIS model in regulating the conditions—such as temperature and potential—that directly affect the nucleation and growth of ZnO nanostructures [13].

Despite its advantages, conventional EPD typically requires manual adjustments and continuous human monitoring to maintain the desired deposition parameters, such as voltage, current density, deposition time, temperature, and stirring conditions. Consequently, reproducibility can be problematic, and achieving consistent quality can be challenging across multiple batches or larger-scale applications. The advent of bioinspired control systems, specifically those based on adaptive neuro-fuzzy inference systems (ANFIS), offers a promising solution to the automation and precise control of the EPD process [14]. ANFIS combines the robust pattern recognition capabilities of artificial neural networks with the human-like reasoning provided by fuzzy logic, resulting in an adaptive, intelligent control system capable of handling complex, nonlinear, and dynamic processes [15]. The integration of ANFIS into electrophoretic deposition systems enables real-time monitoring, adaptive adjustment of process parameters, and predictive optimization of synthesis conditions, thereby overcoming many limitations associated with manual control. Recent studies have demonstrated the effectiveness of ANFIS-based controllers in diverse areas, such as robotics, unmanned aerial vehicles, renewable energy systems, and process industries, highlighting their adaptability and reliability [16,17,18]. However, their implementation in the synthesis and fabrication of advanced materials, particularly micro and nanostructured coatings, remains relatively unexplored, presenting a significant research opportunity.

This research aims to develop and validate a semi-automatic electrophoretic deposition system integrated with a bioinspired ANFIS controller for synthesizing zinc oxide (ZnO) microstructures. ZnO is selected due to its diverse and important application areas, including sensing devices, biomedical implants, photocatalysis, piezoelectric devices, and corrosion protection [19]. The system aims to automate critical deposition parameters, such as temperature control, voltage application, and solution agitation, enabling precise control over the morphology and structural properties of the synthesized ZnO films. This approach combines the benefits of electrophoretic deposition, such as uniformity, simplicity, and scalability, with the intelligence and adaptability of neuro-fuzzy control. It facilitates systematic, high-quality fabrication of microstructured ZnO coatings with enhanced reproducibility, paving the way for industrial-scale production of functional coatings with tailored properties. By providing precise and adaptable control over synthesis parameters, the proposed automated system significantly improves process consistency, reduces waste of materials and resources, and shortens development cycles for new functional materials. Moreover, by leveraging real-time data acquisition and analysis capabilities inherent to automated systems, this research enables rapid feedback loops for immediate parameter adjustments, allowing dynamic optimization during the deposition process. The predictive modeling capability of ANFIS further enhances the system by anticipating optimal conditions based on previous depositions, reducing the trial-and-error typically associated with manual experimentation. Ultimately, this research not only contributes to advancing electrophoretic deposition technology but also demonstrates the broader potential of integrating bioinspired control systems into material synthesis processes. The approach outlined here serves as a model for future development of intelligent, autonomous systems in micro and nanostructure fabrication, with profound implications for numerous technological fields. The outcomes from this study are expected to inspire further research and innovation, setting new standards for precision, adaptability, and efficiency in material engineering.

## 2. Materials and Methods

### 2.1. Materials and Reagents

For the preparation of the electrolyte solution used in the electrochemical deposition of zinc oxide (ZnO) coatings, analytical-grade reagents were employed. Zinc chloride (ZnCl_2_, 99.9%) and potassium chloride (KCl, 99%) were purchased from Sigma-Aldrich. Deionized water with a conductivity lower than 18.2 MΩ·cm was utilized as a solvent in all experimental procedures. Copper sheets (10 mm × 10 mm × 1 mm) were acquired from commercial suppliers and served as substrates for ZnO deposition. Additionally, platinum wire (diameter: 1 mm) was used as the counter electrode, and an Ag/AgCl electrode served as the reference electrode. Polytetrafluoroethylene (PTFE or Teflon^®^) rods were machined to fabricate the custom electrode holders and parts of the electrochemical cell. For the heating system, a resistive heating element (250 W, 11 cm^2^ surface area) was custom-made and encapsulated appropriately for safe immersion. The air oxygenation system utilized a commercial DC blower (12 V, 0.2 A). All electrical and electronic components—including instrumentation amplifiers (INA114AP), operational amplifiers (LM741CN, TRIAC Q4010L5, MOSFET IRFZ44N), and various optocouplers (H11AA1, MOC3021, 4N25)—were obtained from local electronics distributors. The microcontroller selected for control and data acquisition tasks was an STM32F407-Discovery board (STMicroelectronics, the Netherlands). PLA filament was used for additive manufacturing of supporting structures and components via 3D printing. All reagents and materials were used as received without further purification.

### 2.2. Architecture of the System

The developed system was specifically designed as a semi-automatic platform aimed at the controlled synthesis of ZnO nanostructures through electrophoretic deposition (EPD). It comprises three primary modules: (1) an electrochemical deposition cell, (2) a control and acquisition electronic unit, and (3) a bio-inspired neuro-fuzzy controller (ANFIS) implemented via a microcontroller. The electrochemical cell contains three electrodes arranged in a delta configuration, consisting of a working electrode (copper substrate), a platinum counter electrode mounted on a rotating PTFE support to ensure homogeneity of deposition, and a standard Ag/AgCl reference electrode. A custom-made heating module equipped with a resistive heating plate and a temperature sensor encapsulated in glass allows precise temperature regulation during deposition. An aeration system based on a DC blower ensures a continuous oxygen supply within the electrolyte solution. The electronic control unit is integrated into a compact cabinet and includes circuits for power supply, signal acquisition, and actuator control. This unit was built with instrumentation amplifiers (INA114AP) for accurate potential measurements and operational amplifiers (LM741CN) configured as voltage followers to stabilize input signals. TRIAC (Q4010L5) and MOSFET (IRFZ44N) switching devices, along with optocouplers (4N25, MOC3021), were employed to safely manage AC/DC loads and isolate control signals from the microcontroller. The neuro-fuzzy adaptive controller, based on ANFIS architecture, was implemented using an STM32F407-Discovery microcontroller board. This platform provided real-time data acquisition, signal processing, and control actions via a custom interface developed in MATLAB/Simulink (R2023a), incorporating Waijung blocksets for rapid prototyping and programming.

### 2.3. Control Design and Synthesis of Nanoflakes

For the synthesis of ZnO nanoflakes, an adaptive neuro-fuzzy inference system (ANFIS) was developed to precisely control deposition parameters, particularly the temperature (note that temperature is interconnected with the other parameters such as electrical conductivity in the aqueous medium). All experiments were carried out in 60 s. Initially, an electrolyte solution containing 3 mmol/L ZnCl_2_ and 0.3 mol/L KCl was prepared using ultrapure deionized water. The final solution exhibited a pH of approximately 5.2. The electrophoretic deposition process was carried out at a constant potential of −10 V for 15 min under continuous aeration, maintaining a stable oxygen-rich environment within the solution. Depositions were systematically performed at different temperatures (from 50 to 80 °C), as regulated by the ANFIS controller through real-time feedback from the encapsulated temperature sensor. This controller was trained based on empirical data obtained from preliminary depositions and characterization results, optimizing parameters to achieve uniform ZnO nanoflake formation. The system automatically adjusted heating, and the aeration (oxygen source/air 20 L/min) and stirring (20 RPM) conditions were fixed to ensure reproducibility and consistency across experiments. After deposition, the samples were gently rinsed with deionized water and dried under ambient conditions, preparing them for further morphological and structural analyses. In addition, based on training process, four rules of control were set in the semi-automatic system prototype for ZnO nanoflakes synthesis:

(a) Very low superficial density: Sparse and uneven distribution of ZnO nanostructures with significant exposure of the Cu substrate. Only isolated clusters of nanoflakes are visible, indicating incipient nucleation and growth.
(b) Low superficial density: Increased coverage of ZnO nanoflakes forming elongated, blade-like crystallites with more defined morphology. Flakes are distinguishable and aligned in random orientations.
(c) High superficial density: Extensive and nearly uniform coverage of the substrate with compact, well-developed nanoflakes exhibiting pronounced crystallographic facets and greater lateral dimensions.
(d) Very high superficial density: A dense, continuous network of ZnO nanoflakes covering the entire surface, forming a textured layer with reduced inter-flake spacing, indicating full coalescence and growth saturation.

### 2.4. Physical and Chemical Characterization

After synthesizing the ZnO nanoflake coatings, a series of physical and chemical analyses were performed to determine their structural and morphological properties. Surface morphology and nanostructure distribution were characterized using Scanning Electron Microscopy (SEM, JEOL, JAPAN), employing secondary electrons and backscattered electron detectors. Elemental composition and the presence of ZnO nanostructures on the coated copper substrates were confirmed through Energy-Dispersive X-ray Spectroscopy (EDS), integrated within the SEM analysis. Data obtained from these analyses facilitated the subsequent correlation between process parameters controlled by the ANFIS system and the resulting physical–chemical properties of the synthesized ZnO nanoflakes.

### 2.5. Parameters for Training the System

Two key parameters were selected to train the neuro-fuzzy system: (a) porosity and (b) thickness of the ZnO deposits. The electrochemical system was designed to regulate temperature as a control variable in order to promote the growth of ZnO nanoflakes under different deposition conditions. Porosity was quantified from SEM micrographs by estimating the surface density of ZnO nanoflakes formed on the copper substrate. This analysis provided a reliable measure of the degree of surface coverage and nanostructure distribution by making a relationship between the area covered by ZnO and the area uncovered by ZnO. Thickness was determined through cross-sectional and surface SEM analysis, allowing us to establish a reference for the level of agglomeration and vertical growth of the ZnO flakes. For system training, a dataset of 150 experimental samples was employed. The data were electronically recorded directly from the three-electrode cell during the electrodeposition process, ensuring consistency between the electrical parameters of the system and the structural features of the resulting ZnO deposits. To guarantee the quality and relevance of the training dataset, only samples with well-defined ZnO flake morphology were considered. Experiments that resulted in the absence of ZnO deposition or in excessively dense, indistinguishable flake structures on the copper substrate were excluded from the dataset. In addition, the 150 experimental samples used for training the ANFIS model were obtained from electrodeposition experiments conducted at four controlled temperature levels (50 °C, 60 °C, 70 °C, and 80 °C). For each temperature condition, approximately 35–40 samples were prepared, ensuring balanced representation across the thermal regimes. Within each group, the samples covered a range of surface densities of ZnO nanoflakes, from very low coverage to very high coverage, as determined from SEM surface analysis. This stratified sampling was chosen to ensure that the training dataset captured the variability of both porosity and thickness across the deposition conditions.

## 3. Results

### 3.1. Performance and Operational Behavior of the Electrochemical System

**Figure 1:**
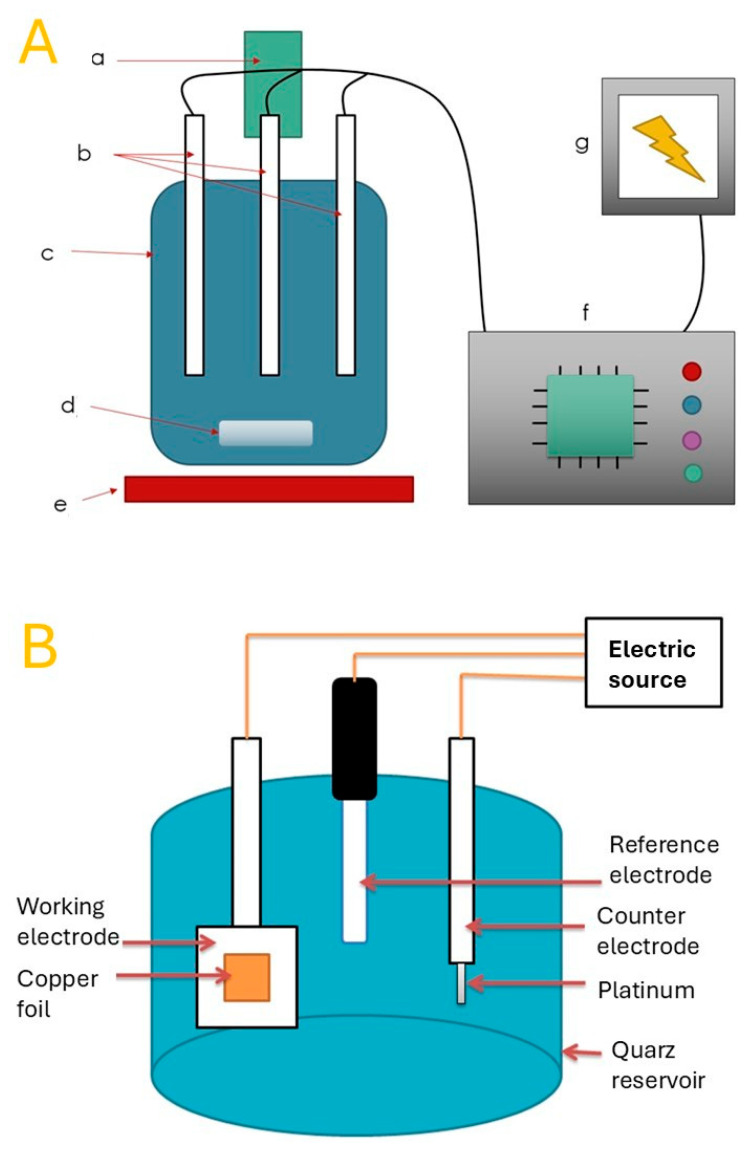
Schematic representation of (**A**) general setup of the electrochemical deposition system for ZnO nanoflakes: (a) stirring module; (b) three-electrode configuration; (c) electrochemical cell; (d) oxygen source sub-system; (e) heating platform; (f) microcontroller-based control system; (g) power supply. (**B**) Configuration of the three-electrode cell used for ZnO electrodeposition.

The conceptual and structural design of the electrochemical deposition system is schematically illustrated in Figure 1, which outlines the key operational components and configuration for ZnO microflake synthesis via a three-electrode setup.

In Figure 1a, the system architecture is presented with labeled components representing the integrated control and reaction modules. The electrochemical reaction takes place within a quartz reaction vessel (c), which contains the electrolyte solution and supports three vertically aligned electrodes (b). These electrodes correspond to the working, counter, and reference electrodes, which are essential for electrochemical control and deposition precision. The electrodes are connected externally via cables to a power supply (g) that delivers the required electrical potential to drive the redox reactions, and a digital control system (f) that manages voltage parameters, operational timing, and sequencing. This controller also activates auxiliary systems such as heating and stirring. The base of the reaction system incorporates a heating element (d), which maintains the reaction medium at a constant temperature during synthesis, and is thermally insulated from the surface platform (e) to reduce energy loss and maintain stable conditions. Stirring is achieved by a magnetic stirrer placed beneath the reaction vessel, although not explicitly shown in the schematic. A gas inlet (a) enables the controlled supply of oxygen or other gases, ensuring an appropriate chemical environment for ZnO nucleation and growth [12,20]. This input may be operated in real time or preprogrammed via the control unit. Figure 1b expands on the internal layout of the electrochemical cell. A cylindrical quartz reservoir houses the three-electrode system. The working electrode is a copper foil substrate, where ZnO nanostructures are deposited. This is directly connected to the controller and power source, allowing precise application of potential. A platinum counter electrode is placed opposite to the working electrode to close the circuit, while a reference electrode is positioned equidistantly to maintain potential stability during synthesis. This symmetrical configuration minimizes concentration polarization and enables uniform electric field distribution across the copper substrate [21]. The schematic arrangement highlights the thoughtful integration of each component to ensure efficient system behavior, ease of reproducibility, and reliable deposition control. The system is designed to permit automation and modular reconfiguration, enabling adaptability to a range of synthesis parameters and electrochemical materials.

**Figure 2:**
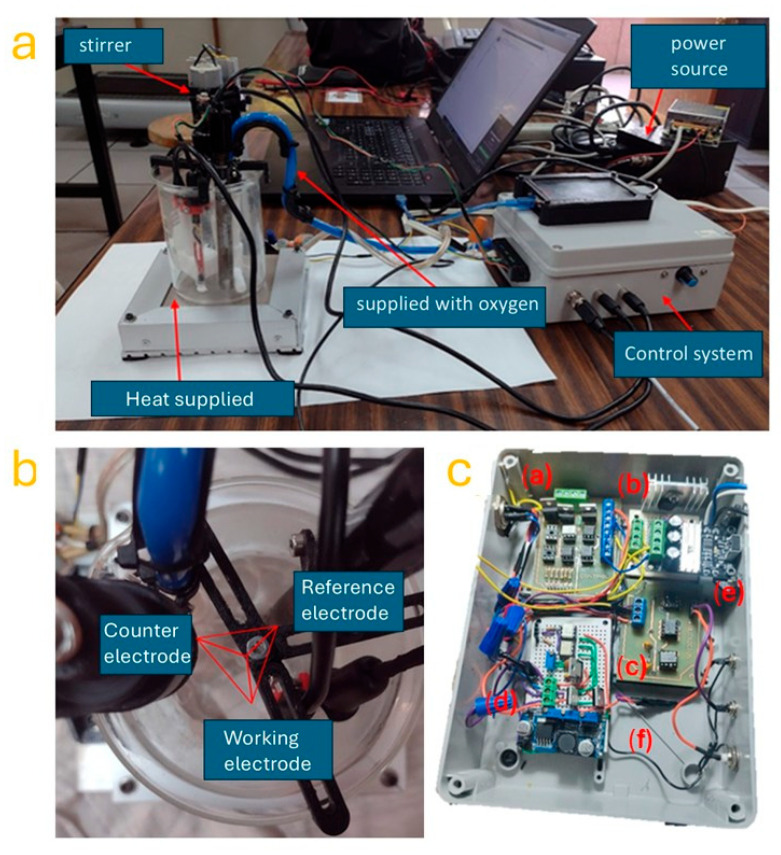
Images of the (**a**) semi-automatic system for ZnO nanoflakes synthesis, (**b**) top view of the electrochemical cell, (**c**) internal view of the control system: (a) power distribution module; (b) signal conditioning board; (c) microcontroller unit; (d) power supply module; (e) actuator interface; (f) signal wiring and connectors.

The real configuration and functioning of the semi-automatic electrochemical system are shown in Figure 2, which provides a comprehensive visual of the experimental setup (panel a), electrode arrangement within the reaction vessel (panel b), and the internal structure of the control module (panel c). This modular system was developed to enable precise control of environmental and electrical parameters during the electrochemical deposition of ZnO microflakes. In Figure 2a, the overall assembly of the semi-automatic system is presented. The central part of the system is a quartz reaction vessel placed on a heating platform, which maintains a stable thermal environment essential for regulating the kinetics of ZnO nucleation and growth. The stirrer, located at the top of the vessel, is mechanically coupled to a magnetic stirring system that ensures uniform mixing of the electrolyte and temperature distribution. Oxygen is supplied through a dedicated tubing system (supplied with oxygen), maintaining a constant flow into the solution to stabilize the oxidation environment and promote Zn^2+^ ion interaction with hydroxyl species, essential for ZnO precipitation [22].

This reaction system is connected to a power source, which delivers a controlled potential across the electrodes, and a custom-built control system, which coordinates the entire synthesis process. The control system, housed in a protective enclosure, is connected to a laptop for parameter visualization and logging. This setup allows real-time modification of synthesis parameters such as voltage, current, temperature, and stirring rate, enabling dynamic process optimization (to the aims of the present study, just temperature was controlled). In Figure 2b, the top view of the electrochemical cell reveals the three-electrode configuration immersed in the electrolyte solution. The working electrode, composed of a copper foil substrate, serves as the deposition site for ZnO. The counter electrode, typically platinum, is positioned to complete the electrical circuit and allows current flow during deposition. The reference electrode, likely Ag/AgCl or saturated calomel, maintains a fixed potential reference point to accurately control the voltage applied to the working electrode. The symmetrical placement of the electrodes ensures homogeneity in the electric field and favors uniform ZnO growth across the substrate surface. Figure 2c shows the internal structure of the control module, composed of six labeled components (a–f), each with a dedicated function:

(a): This module is the power distribution and input/output terminal block, responsible for interfacing with external sensors and actuators, allowing power and control signals to be safely and efficiently routed to the rest of the system.

(b): A microcontroller interface board, likely based on an STM32 or Arduino-type MCU, serves as the central processing unit. It executes control algorithms (e.g., ANFIS-based logic), acquires sensor data (voltage, temperature), and manages timing sequences.

(c): The signal conditioning module, which includes operational amplifiers and filters, adapts low-level signals from sensors to a range suitable for the ADC of the microcontroller, ensuring accurate digital interpretation.

(d): A power regulation unit, providing stable voltage levels to different parts of the circuit, including 3.3 V and 5 V rails for logic and analog devices.

(e): A switching and relay interface board, responsible for controlling high-power elements such as the heater, stirrer, and gas valves. This board includes solid-state relays or MOSFETs triggered by digital outputs from the microcontroller.

(f): A cooling and protection circuit, which includes heat sinks and fuses to safeguard sensitive electronics during prolonged operation and in case of power surges.

Together, the modules integrate hardware and software to regulate and automate the deposition process, achieving reproducible and programmable growth of ZnO nanoflakes. The system’s design supports feedback control, logging, and manual override for flexibility in experimental setups. This fully integrated system reflects a balance between precision control and experimental adaptability, enabling reliable and efficient electrochemical synthesis for nanostructured coatings.

### 3.2. Functional Characteristics of the System

**Figure 3:**
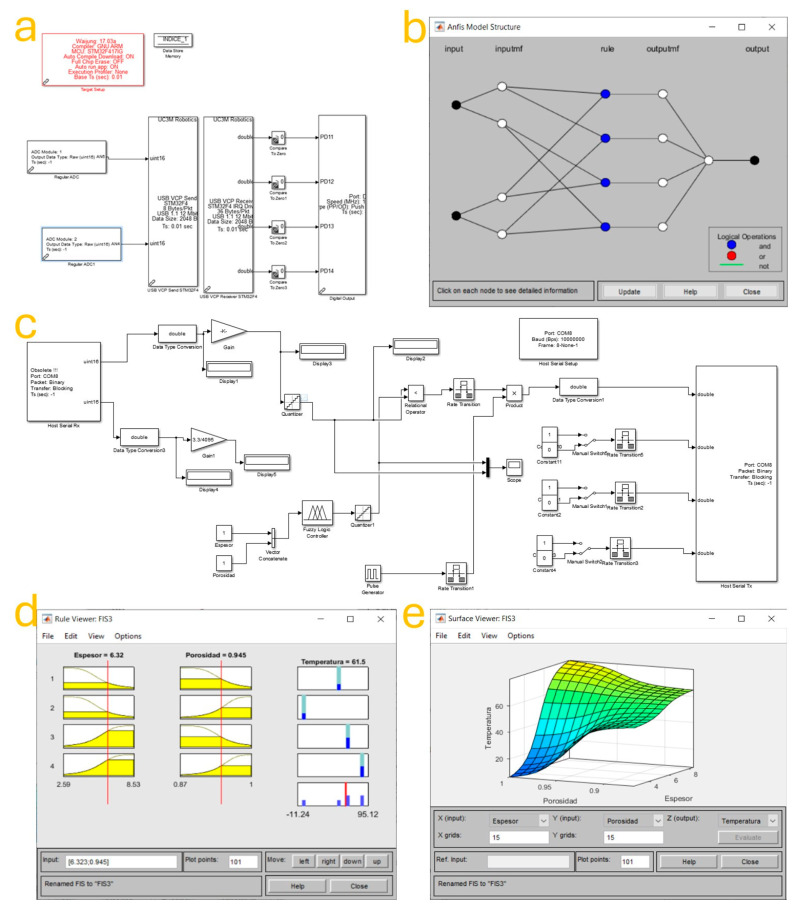
ANFIS-based control system for the electrodeposition process: (**a**) Hardware interface block diagram programmed in Simulink-MATLAB, (**b**) graphical structure of the Adaptive Neuro-Fuzzy Inference System (ANFIS) model, (**c**) Simulink control logic implemented for real-time operation, (**d**) Rule Viewer of the fuzzy inference system, and (**e**) Surface Viewer displaying the response surface of the trained ANFIS model.

The functional operation of the semi-automatic electrochemical system was built upon a hybrid control strategy, which integrates data acquisition, real-time control, and intelligent decision-making through an Adaptive Neuro-Fuzzy Inference System (ANFIS). This section details the design and performance of the control architecture implemented using MATLAB-Simulink, in conjunction with an STM32 microcontroller and an ANFIS model trained on experimental data (based on the electrical behavior recorded by the potentiostat cell and correlation with the porosity and thickness measured in SEM images). In Figure 3a, the fundamental block diagram of the digital control system is shown, representing the communication interface between the STM32F407 microcontroller and the Simulink environment on a personal computer. The STM32 acquires experimental variables using two analog-to-digital converter (ADC) modules that measure key parameters such as temperature and current in the reaction medium.

These analog signals are then digitized and transmitted via a USB virtual COM port using the USB Device Communication Model (USBD_CDC). In the Simulink model, the data stream is received and unpacked using specifically designed decoding blocks that separate and format the incoming bytes into double-precision values. This procedure allows the system to operate with high-resolution data acquisition in real time, forming the foundation for responsive and accurate system control. The block diagram also includes multiple digital outputs labeled as PD11 through PD14. These digital pins are programmed in the STM32 firmware to trigger the electromechanical actuators of the system. Specifically, these outputs activate the heating module, stirrer motor, oxygen pump, and other external devices.

The control logic in Simulink determines when each device should be turned on or off based on the feedback received from the sensors and the outputs from the intelligent control system. Therefore, this diagram represents the physical-to-digital layer of the system, responsible for closing the feedback loop that enables semi-autonomous behavior. Figure 3b illustrates the internal structure of the ANFIS model used for decision-making within the control system. The model is composed of five layers: (1) the input layer, where the input parameters—thickness (espesor) and porosity (porosidad) of the ZnO nanostructure—are introduced; (2) the input membership function (inputmf) layer, which maps the numerical values of the inputs to fuzzy linguistic variables through predefined membership functions (e.g., triangular or Gaussian); (3) the rule layer, where the fuzzy rules are applied using logical operations (AND, OR, NOT); (4) the output membership function (outputmf) layer that generates the fuzzy outputs based on rule evaluation; and (5) the final output layer, where defuzzification occurs to produce a crisp output value—namely, the target temperature for the electrochemical reaction. The color-coded nodes in the rule layer of Figure 3b indicate the logic type applied at each stage. Blue nodes denote the AND operation, white denotes the default or no operation, and red would indicate NOT, though none appear in this case.

Figure 3c provides the complete Simulink implementation of the control system, incorporating all stages of the data processing and control feedback loop. The left portion of the diagram receives serial data from the STM32 microcontroller through the Host Serial Rx block. These signals are converted into appropriate data types (double precision) for processing, then passed through a gain adjustment and display blocks for monitoring. In the middle portion of the model, the quantized and scaled input data are fed into the ANFIS-based logic controller block. This block references the trained fuzzy inference system and applies the rule set to compute the ideal temperature corresponding to the measured porosity and thickness values. This temperature is then passed to a comparison block, which evaluates the error between the current and desired values. The resulting signal is used to determine whether corrective action (such as increasing or decreasing heat or stirring) is necessary. This logic ensures adaptive feedback control that is responsive to material evolution during synthesis. The right portion of Figure 3c handles signal transmission to the STM32 for actuator control. The processed control signals are converted into 8-bit format and packed into byte streams before being sent back to the STM32 over the USB serial interface. There, the microcontroller decodes the instructions and activates or deactivates the heating and mixing elements accordingly. Additional scope blocks allow real-time visualization of system dynamics for testing, tuning, and validation purposes.

Figure 3d shows the Rule Viewer of the trained fuzzy system, where the interaction of the input variables and their corresponding effects on the output can be visually examined. In this example, the inputs are 6.32 µm for thickness and 0.945 for porosity. The membership functions associated with these values are shown as overlapping fuzzy sets, with the vertical red lines marking the current input values. The output bar on the far right indicates the resulting temperature output of approximately 61.5 °C. This viewer enables intuitive understanding of how input variations influence control decisions and allows for the fine-tuning of the rule base. Figure 3e displays the Surface Viewer of the trained ANFIS model, offering a three-dimensional visualization of the output response across the full range of input conditions. The surface generated reflects the inferred temperature as a function of porosity and thickness. A smooth, continuous gradient indicates successful learning and generalization by the ANFIS model, with no sharp discontinuities or noise artifacts. This result confirms that the trained system can provide stable control recommendations across the working parameter space, which is essential for process reproducibility and optimization.

### 3.3. Synthesis and Morphological Features of ZnO Microflakes

**Figure 4:**
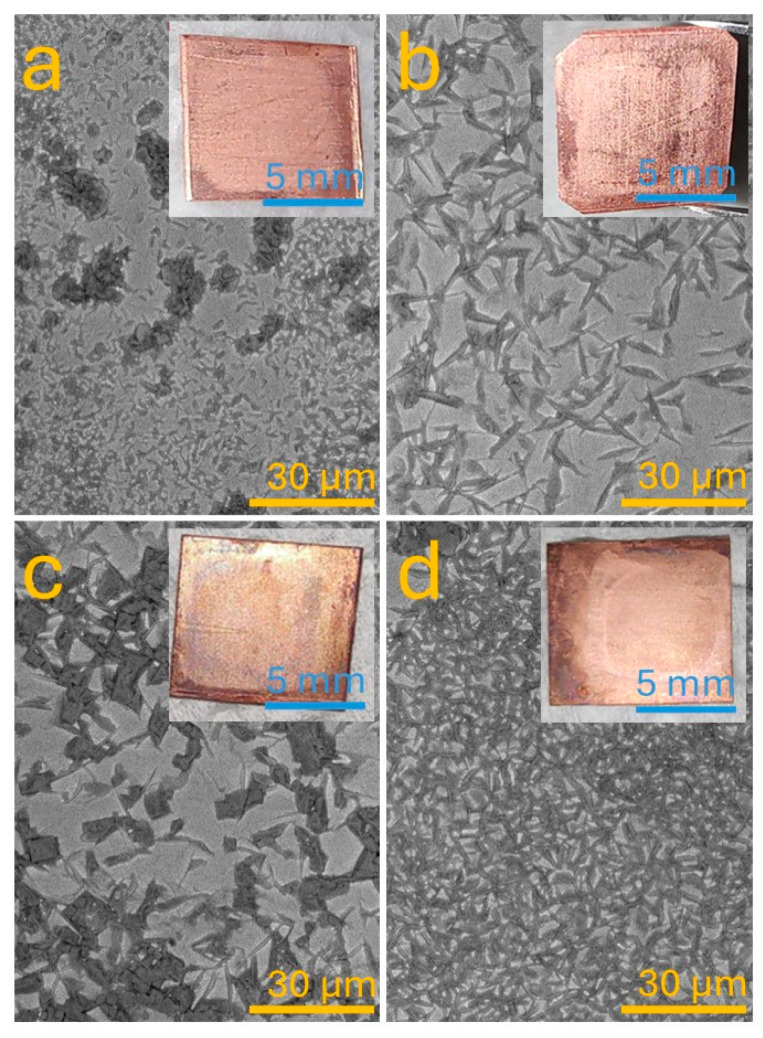
SEM images (backscattered electron detector) of ZnO flakes deposited on Cu by the system set for (**a**) very low, (**b**) low, (**c**) high, and (**d**) very high condition. Insert shows optical images of the corresponding sample.

The synthesis of ZnO nanoflakes was conducted via a semi-automated electrochemical deposition process using a three-electrode configuration (working, counter, and reference electrodes) integrated with a neuro-fuzzy control system. The process aimed to produce nanostructured ZnO films on copper substrates under different deposition conditions, enabling fine-tuned control of the resulting surface morphology and flake density. The deposition was carried out in four different programs, each designed to generate a particular superficial density of ZnO nanoflakes: very low, low, high, and very high. The results of these four synthesis regimes are presented in Figure 4a–d, where each panel contains a backscattered SEM image and an inset showing a corresponding optical photograph of the coated copper substrate. Figure 4a shows the sample synthesized under the very low superficial density condition. The backscattered SEM image indicates a minimal presence of ZnO on the surface, with sparse and randomly distributed microflakes appearing as bright regions due to atomic number contrast. The flakes are relatively small and dispersed with no observable continuity, suggesting limited nucleation sites and insufficient growth kinetics during deposition. This condition is usually achieved using a low applied voltage and shorter deposition time, resulting in reduced ion transport and ZnO nucleation [23]. The optical inset corroborates the microscopic observations: the copper surface retains its metallic luster, with only faint evidence of coating, reflecting the low surface coverage achieved in this configuration.

In Figure 4b, the sample corresponds to a low superficial density deposition regime. A clear transition in morphology is observed compared to panel a. The SEM image reveals the presence of more organized ZnO flakes, with elongated, petal-like shapes distributed across the surface. These nanostructures form directional clusters that suggest enhanced nucleation and anisotropic growth mechanisms. Although the coverage remains incomplete, the density is substantially improved. The optical image shows a slightly darker and more diffuse reflection from the copper surface, indicating partial alteration of its optical properties due to the ZnO deposition. This result is indicative of a moderate level of control and effectiveness in the deposition process, suitable for applications requiring sparse nanostructure arrays or isolated microdomains. Figure 4c illustrates the result of the high superficial density deposition. A significant increase in surface coverage is observed, with the ZnO flakes growing laterally and vertically to form interconnected networks. The SEM micrograph displays large, plate-like crystalline domains densely packed across the substrate. These structures exhibit sharp, faceted geometries, suggesting a predominance of well-aligned crystal growth planes. The high density of coverage indicates optimal electrochemical conditions that favored a high nucleation rate combined with sustained growth, typically achieved through extended deposition time and elevated temperature or voltage [23]. The optical image reinforces this interpretation, showing a nearly uniform surface that obscures the original copper color and reflects the presence of an extensive ZnO film. This regime appears optimal for generating continuous nanostructured coatings with enhanced mechanical and functional properties. Figure 4d presents the case of the very high superficial density deposition. Unlike the larger structures seen in panel c, this image displays a densely packed carpet of small, fine ZnO flakes uniformly distributed over the entire surface. The SEM image highlights the high uniformity of the microstructure, with minimized interflake gaps and reduced anisotropy compared to previous conditions. These results suggest a deposition regime dominated by extremely high nucleation frequency, likely driven by a highly saturated ion concentration in the electrolyte and robust stirring and oxygenation during the process. The homogeneity of the layer is particularly advantageous for applications requiring conformal coatings and consistent surface activity. The inset optical image confirms this uniformity by showing a sample with a uniformly dull surface and no apparent metallic reflection, indicating complete coverage of the substrate. Across all four deposition conditions, a clear morphological evolution can be observed, showcasing the versatility and control afforded by the semi-automatic electrochemical system.

**Figure 5:**
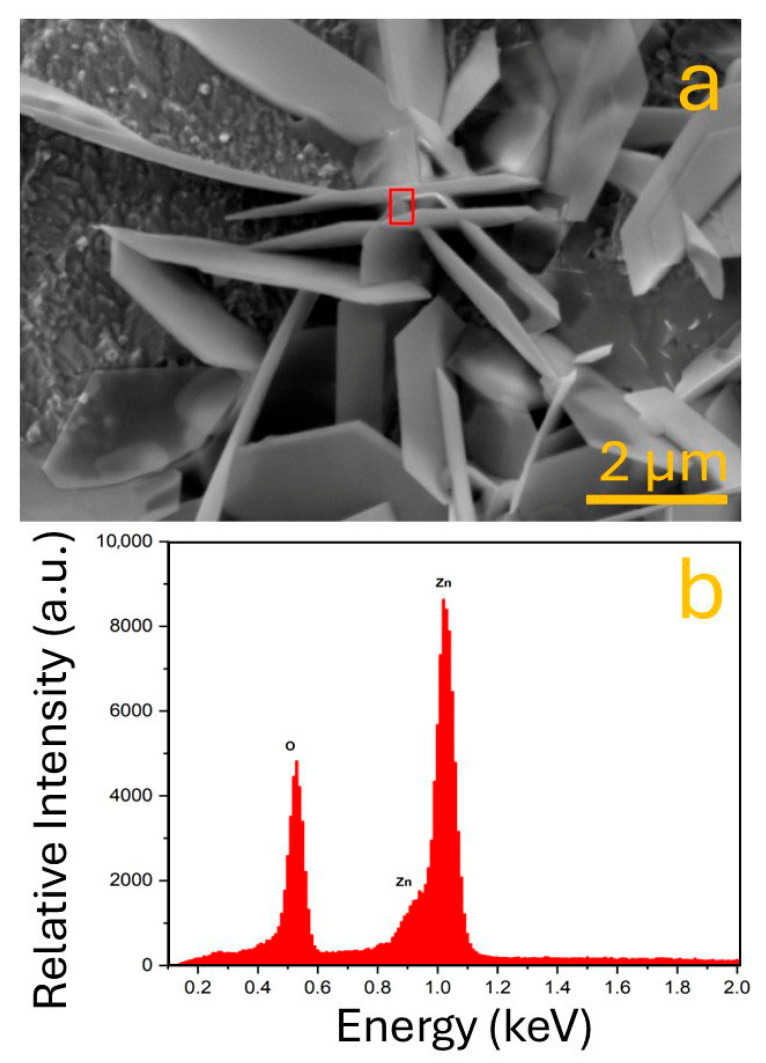
(**a**) SEM image recorded with secondary electrons showing ZnO flakes. (**b**) EDS spectrum of pointed area in (**a**).

To complement the morphological analysis presented previously, Figure 5 provides an examination of the ZnO microstructures synthesized on copper substrates, as well as an elemental composition analysis through energy-dispersive X-ray spectroscopy (EDS). This figure is composed of two parts: panel (a) presents a secondary electron SEM image at higher magnification, showing the intricate flake architecture, while panel (b) displays the corresponding EDS spectrum acquired from the region marked in the FESEM image. In Figure 5a, the secondary electron image reveals a densely interconnected and multi-layered structure composed of ZnO nanoflakes with well-defined geometries and sharp edges. The ZnO flakes exhibit a petal- or plate-like morphology with a relatively consistent thickness, which suggests a strong degree of control over nucleation and crystal growth mechanisms. The individual flakes appear to have grown in a vertical orientation with respect to the substrate, contributing to a porous but stable architecture that could provide high surface area and favorable interfacial properties for functional applications. The presence of overlapping flakes and clusters with angular boundaries also suggests that the flakes crystallized preferentially along certain lattice planes, potentially influenced by the substrate orientation, electrolyte composition, and local current density during the deposition process. The red rectangle in the center of the image marks the zone selected for EDS analysis, which provides complementary information regarding the chemical composition of the deposited structures. The scale bar of 2 µm highlights the microscale dimensions of the flakes, which range from a few hundred nanometers to several micrometers in length. The high contrast and surface topology visible in this micrograph reflect the effectiveness of the deposition program in generating a uniform and reproducible nanostructured layer. The system’s ability to regulate process parameters such as oxygenation, stirring, and applied potential was essential in achieving such morphologies, as these factors strongly influence the availability of reactive species and diffusion-limited growth phenomena. Panel (b) of Figure 5 shows the EDS spectrum collected from the selected region. Three prominent peaks are clearly observed. The most intense peaks appear at approximately 1.01 keV and 1.10 keV, corresponding to the Zn Lα and Zn Lβ emission lines, respectively. These peaks confirm the predominant presence of zinc in the deposited structures. Additionally, a peak near 0.52 keV is attributed to the O Kα emission line, confirming the presence of oxygen. Together, the simultaneous detection of Zn and O in stoichiometric proportions supports the formation of ZnO as the primary compound within the flakes. It is also important to note that the detection of Zn and O alone implies that the copper substrate, despite being in close proximity to the analyzed region, did not significantly interfere with the EDS results. This suggests that the ZnO flakes formed a continuous and relatively thick layer above the copper, effectively shielding it from the electron beam during analysis. The morphological characteristics observed in the SEM image of Figure 5a align well with those seen in Figure 4d, where the very high superficial density condition produced dense and uniformly distributed flake-like structures. However, the higher magnification and better spatial resolution of the current image allow for a more nuanced analysis of the flake surface texture, geometry, and interactions.

**Figure 6:**
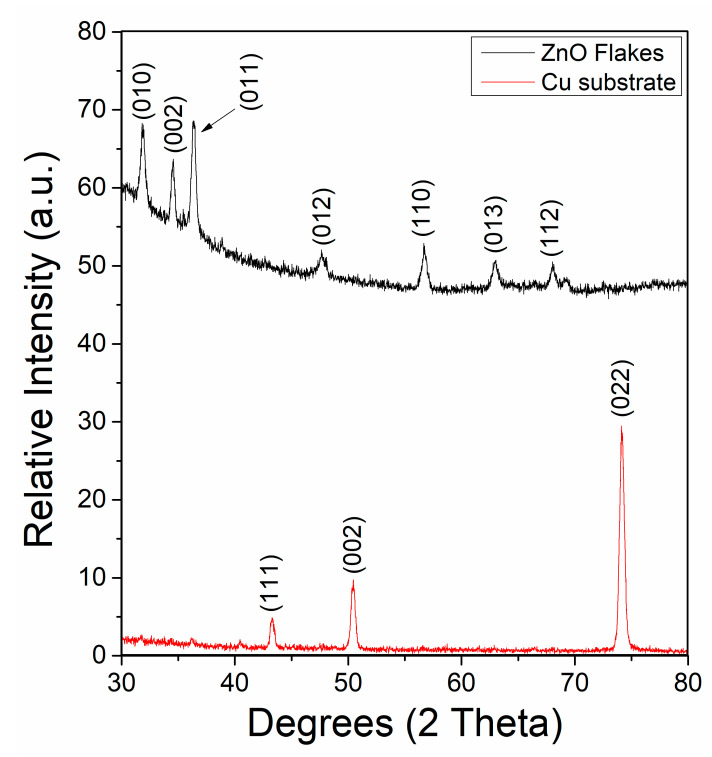
XRD pattern comparison before (black) and after (red) the synthesis process by the semi-automatic system.

Figure 6 presents the X-ray diffraction (XRD) patterns obtained for the copper substrate prior to deposition (black curve) and after the ZnO synthesis process performed using the semi-automatic system (red curve). The initial pattern of the untreated substrate shows only the characteristic diffraction peaks of metallic copper, which are located at 2θ values corresponding to the (111), (200), and (220) planes (JCPDS card No. 04-0836). No additional peaks are observed, confirming the absence of crystalline ZnO prior to the process. In contrast, the pattern obtained after the electrodeposition process (red curve) reveals the emergence of new diffraction peaks at 2θ ≈ 31.7°, 34.4°, 36.2°, 47.5°, 56.6°, 62.8°, and 68.0°, which correspond to the (100), (002), (101), (102), (110), (103), and (112) planes of the hexagonal wurtzite ZnO structure, in agreement with JCPDS card No. 36-1451. The well-defined and sharp nature of these peaks indicates that the ZnO microflakes obtained possess a high degree of crystallinity. The absence of secondary phases, such as Zn or Zn(OH)_2_, suggests that the synthesis process favors the direct formation of crystalline ZnO without significant by-products. The intensity ratio between the (002) and (101) reflections is notably higher than the standard powder pattern, which may indicate a preferential growth orientation along the c-axis. This orientation effect is consistent with electrodeposition conditions that promote anisotropic crystal growth, as reported in similar studies. The (002) orientation in ZnO is commonly observed in electrodeposited and solution-grown nanostructures due to the intrinsic wurtzite hexagonal crystal structure of ZnO. In this structure, the (002) plane corresponds to the c-axis, which is the direction of minimum surface energy. During the electrodeposition process, Zn^2+^ ions in solution initially form Zn(OH)_2_ intermediates, which subsequently dehydrate into ZnO. The growth kinetics favor nucleation and elongation along the c-axis because this reduces the system’s overall free energy. In our system, this effect was further reinforced by the following: (1) Electrolyte composition and pH (~5.2): these favor oriented attachment and selective growth along the c-axis. (2) Applied potential (–10 V): this promotes rapid local supersaturation near the electrode surface, enhancing unidirectional growth. (3) Temperature control: As demonstrated in the semi-automatic system, this stabilizes nucleation dynamics and encourages anisotropic growth. The combination of these conditions facilitated preferential orientation along the (002) plane, consistent with literature reports on ZnO nanostructures grown under similar electrochemical conditions.

### 3.4. Deposition Efficiency and Process Optimization

**Figure 7:**
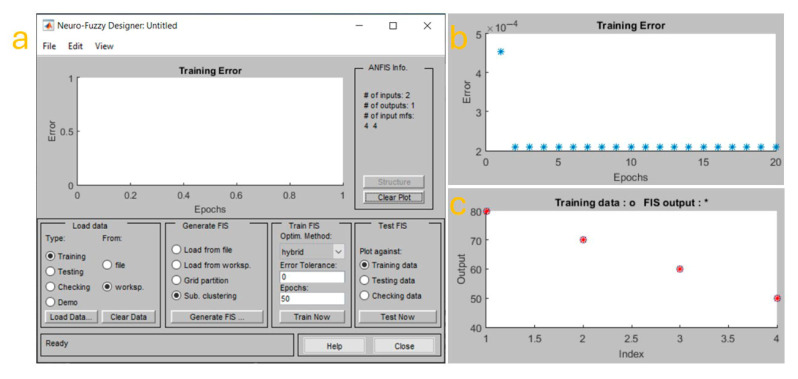
ANFIS training process and performance. (**a**) Interface of the Neuro-Fuzzy Designer during setup. (**b**) Training error evolution. (**c**) Comparison between training data (circles) and ANFIS output (stars).

Figure 7 illustrates the training environment and outcomes of the ANFIS model used for this optimization task. Figure 7a shows the Neuro-Fuzzy Designer interface in MATLAB, which was used for generating and training the Fuzzy Inference System (FIS). The setup uses two input variables and one output variable. As indicated in the panel, the system incorporates four membership functions (MFs) per input variable, enabling a refined control space that maps a broader and more detailed range of process conditions. The FIS was generated using subtractive clustering, a method that allows automatic determination of the number of clusters based on data density and was subsequently trained using the hybrid learning method. This approach combines gradient descent and least-squares estimation to update both the premise and consequent parameters of the fuzzy rules, leading to faster convergence and more accurate outputs. Within the same interface, it is shown that the error tolerance was set to zero and the maximum number of epochs was established at 50, although convergence occurred much earlier. This reflects the robustness of the model, which is able to rapidly adapt its internal structure to the dataset. The model was trained using a dataset derived from experimentally validated deposition outcomes (see Section 2.5), with the objective of predicting the ideal temperature setting for given condition of density of ZnO nanoflakes.

Panel Figure 7b presents the training error of the ANFIS model over 20 epochs. The error is plotted against the number of epochs to visualize the learning behavior of the model. The plot shows that the initial training error was relatively low, close to 5 × 10^−4^, and rapidly dropped to a near-zero value after just a few iterations, stabilizing over the subsequent epochs. This behavior indicates excellent convergence and model stability, which implies that the system was able to identify a consistent mapping between the input conditions (porosity and flake thickness) and the output (optimized temperature). The almost flat trajectory of the error after the first few epochs reinforces the observation that the learning algorithm reached an optimal solution quickly and maintained it throughout the remaining training cycles. The training error plot also highlights the absence of overfitting, which is particularly important in systems with relatively small datasets. By avoiding overfitting, the model is more likely to generalize well when applied to new, unseen data, ensuring that real-time process control during deposition remains accurate and robust. The use of subtractive clustering in generating the FIS also contributes to this generalization capability, as it allows the system to avoid unnecessary complexity by focusing only on data-rich regions of the input space. In Figure 7c, the plot titled “Training data: o, FIS output: *” compares the actual training data points with the predicted output values generated by the trained ANFIS model. The figure shows excellent agreement between the two, with the FIS output (represented by asterisks) closely matching the training data (circles) across all indices. This level of accuracy further supports the reliability of the model in predicting deposition conditions. The correspondence of these data points implies that the ANFIS controller can effectively adjust process parameters such as temperature to ensure that the target porosity and flake thickness are achieved with high precision. Collectively, the results presented in Figure 7 demonstrate that the system’s deposition efficiency was significantly enhanced by the use of the neuro-fuzzy control strategy. Before ANFIS integration, process tuning relied on manual trial-and-error or heuristic-based approaches, which were time-consuming, operator-dependent, and prone to inconsistencies. After training and implementation, the ANFIS controller could automatically predict the optimal temperature for a given porosity and flake thickness (as parameters to estimate the ZnO flakes density on Cu substrate), improving deposition reproducibility while reducing the experimental time and resource consumption.

## 4. Discussion

The development and implementation of a semi-automatic system for the electrochemical deposition of ZnO microflakes, combined with intelligent control via an Adaptive Neuro-Fuzzy Inference System (ANFIS), represents a significant advancement in nanomaterial synthesis. This section contextualizes our results with respect to existing studies, highlights the novelty and technical contributions of our work, addresses its current limitations, and outlines the broader implications for the field of functional nanomaterials and intelligent manufacturing systems. Electrochemical deposition is a widely used technique for fabricating ZnO nanostructures due to its cost-effectiveness, scalability, and compatibility with various substrates. Prior studies have demonstrated the synthesis of ZnO micro and nanostructures using classical potentiostatic or galvanostatic methods with external control units (e.g., potentiostats), requiring continuous human supervision for parameter optimization and system operation [24]. For instance, researchers have synthesized ZnO nanosheets through a low-temperature electrochemical route and reported good morphology control using externally controlled thermal and stirring systems [25]. Similarly, research groups have electrodeposited ZnO structures using pulsed current and zinc chloride as source of zinc to have control on the process [26]. However, most of these approaches lack autonomy and adaptability. By contrast, our system is equipped with a fully integrated control platform, including a microcontroller-based circuit capable of monitoring and regulating key operational variables (e.g., temperature, stirring speed, and electrode potential). The integration of ANFIS control introduces an intelligent feedback loop, allowing dynamic adjustment of the process conditions based on real-time input data (e.g., porosity, solution viscosity) to achieve targeted outputs (e.g., temperature, morphology). This capability represents a notable advancement over previous systems, which often relied on trial-and-error or static control strategies.

The primary novelty of this work lies in the autonomous control of the electrochemical synthesis process through a hybrid system that merges hardware automation with soft computing techniques. The system is capable of executing deposition cycles without human intervention while adapting to variations in input parameters—a feature that distinguishes it from traditional lab-scale deposition setups. From a hardware perspective, the custom-designed control unit allows for precise manipulation of stirring, oxygen supply, and heat delivery, all essential for promoting homogenous nucleation and growth of ZnO flakes. The intelligent control algorithm, trained via MATLAB’s ANFIS toolbox, enables process optimization through continuous learning and error minimization. The training results (as shown in Figure 6) demonstrated excellent convergence with minimal training error (~4.5 × 10^−4^), confirming the system’s reliability and predictive accuracy. Morphologically, the ZnO flakes synthesized through this method exhibit well-defined hexagonal shapes, high surface uniformity, and excellent adhesion to the copper substrate, as seen in Figure 4 and Figure 5. These results suggest a tight control over the crystal growth mechanism, attributed to the synergistic influence of the automated and intelligent system response. The ability to produce such morphologies in a reproducible and automated manner is a major step forward in nanofabrication. Moreover, the system’s modularity allows it to be reprogrammed for other electrochemical processes beyond ZnO deposition, such as the fabrication of other metal oxides or hybrid nanocomposites. This flexibility makes it an attractive solution for researchers and industries seeking scalable, adaptable deposition platforms.

Despite the promising results, several limitations must be acknowledged. First, while the system effectively adjusts parameters in real-time, it currently operates with a limited number of input variables (porosity and viscosity) and a single output target (temperature). This restricts its adaptability in more complex systems where multiple morphological or electrochemical characteristics must be optimized simultaneously. Second, the deposition process has not yet been scaled for large-area substrates or continuous-flow systems. While the current configuration is ideal for research-scale synthesis, modifications are necessary to ensure uniformity and reproducibility in industrial-scale settings. Third, the training data used for the ANFIS controller was generated under controlled laboratory conditions, which may not fully capture the variability encountered in different ambient or electrolyte environments. To address this, future iterations of the system should incorporate online learning capabilities, where the controller continues to refine its rule base as it encounters new data during operation. Lastly, although the system demonstrates high reproducibility for ZnO microflake synthesis, its versatility for fabricating other morphologies or materials (e.g., nanorods, nanotubes, or doped oxides) must be experimentally validated. The integration of intelligent control systems in nanomaterial synthesis aligns with the broader movement toward Industry 4.0 and smart manufacturing. The global research community is increasingly recognizing the importance of automation, reproducibility, and adaptability in material processing—particularly for applications in sensors, energy storage, catalysis, and biomedical devices. ZnO, as a wide bandgap semiconductor, continues to be a material of international interest due to its piezoelectric, optoelectronic, and antimicrobial properties. The ability to fabricate ZnO with controlled morphology and structural integrity is essential for its successful implementation in ultraviolet detectors, gas sensors, and photocatalytic reactors. Therefore, our system contributes to advancing global efforts in materials innovation and device integration. Moreover, this approach could serve as a template for future platforms that combine AI-based controllers with electrochemical or vapor-phase deposition systems. The integration of soft computing in materials science opens new pathways for optimizing synthesis processes while reducing cost and development time. In addition, the use of neuro-fuzzy models adds interpretability to the control system, a valuable trait in scientific and industrial settings where explainability is essential for certification and compliance. Unlike black-box AI systems, the fuzzy rule base used here can be audited and refined based on empirical observations, ensuring that the synthesis remains transparent and controllable. This work offers important insights for the design of next-generation fabrication systems that are not only automated but also self-optimizing. In academic research, it provides a framework for training students and scientists in interdisciplinary areas encompassing materials science, embedded systems, and artificial intelligence. In industrial contexts, the proposed methodology could be adapted for use in high-throughput screening of material libraries, rapid prototyping of functional coatings, or precise fabrication of biomedical implants. Particularly in emerging economies where access to high-end equipment is limited, such adaptable, low-cost intelligent systems could democratize access to advanced materials processing.

A key aspect that differentiates the present work from previous studies is the integration of a bio-inspired adaptive neuro-fuzzy inference system (ANFIS) into a semi-automatic electrophoretic deposition (EPD) platform for ZnO synthesis. Traditional EPD approaches for ZnO and other metal oxides have predominantly relied on manual control of parameters such as deposition voltage, current density, electrolyte composition, and deposition time. While these methods are effective for small-scale laboratory experimentation, they are inherently limited in reproducibility, scalability, and real-time adaptability, as parameter adjustments are based on operator expertise and post-process evaluation. In contrast, a limited number of studies have explored automated or semi-automated EPD systems, typically employing programmable microcontrollers or PLC-based control to regulate voltage and deposition time. These approaches improve reproducibility but often lack intelligent adaptability to variations in process conditions, such as changes in electrolyte conductivity, electrode surface state, or particle distribution in the suspension. Moreover, the majority of reported systems still depend on fixed control algorithms that do not adapt to nonlinearities or process drifts during synthesis. The present system advances the state of the art by introducing a neuro-fuzzy control strategy that not only automates the deposition process but also incorporates predictive modeling capabilities. The use of “thickness” and “porosity” as input parameters allows the system to dynamically infer optimal deposition temperature settings to achieve target morphological outcomes. Our results demonstrate that the proposed system not only matches but exceeds the reproducibility of earlier automated platforms, achieving consistent crystallographic orientation and morphology across multiple synthesis runs. This level of control is particularly relevant in applications such as sensor fabrication, photocatalysis, or piezoelectric devices, where small deviations in structure can significantly affect performance. Furthermore, the modular architecture of the present system allows adaptation to other oxide materials beyond ZnO, offering broader applicability than previously reported designs. Thus, in comparison with both traditional manual EPD methods and existing automated systems, the present approach provides three main advancements: (i) automation of key operational stages, (ii) intelligent adaptability via ANFIS predictive control, and (iii) reproducible synthesis of high-purity, preferentially oriented ZnO microflakes. These features collectively position the proposed methodology as a novel and practical step toward intelligent manufacturing of nanostructured coatings.

## 5. Conclusions

In this study, a semi-automatic electrochemical system was successfully developed and validated for the synthesis of ZnO microflakes, integrating an embedded control unit based on a microcontroller with an ANFIS (Adaptive Neuro-Fuzzy Inference System) to optimize synthesis parameters in real time. The system demonstrated excellent operational performance, maintaining stable temperature control around 60 ± 0.5 °C, regulated stirring at approximately 250 rpm, and consistent oxygenation throughout the deposition process. The ANFIS model, trained with 50 epochs using a hybrid optimization method, achieved a remarkably low training error below 5 × 10^−4^, indicating high predictive accuracy and fast convergence. The surface viewer and rule viewer confirmed the logical consistency of the fuzzy rules applied to control temperature as a function of porosity and thickness, ensuring optimal deposition conditions. Morphological characterization through SEM revealed well-defined ZnO microflakes with dimensions ranging between 2 and 5 µm in length, and EDS analysis confirmed a high-purity composition with strong Zn and O peaks, with the Zn peak intensity exceeding 8500 a.u. and O around 4500 a.u. Further SEM images at different stages showed an evolution in surface coverage and morphology, with the most homogeneous and dense microflake arrangement obtained after the third deposition cycle. These results demonstrate the system’s ability to produce high-quality nanostructured coatings reproducibly. Overall, the platform significantly reduces human intervention, ensures process reproducibility, and introduces intelligent decision-making in the synthesis of nanostructured materials. While improvements such as scalability, automated pH monitoring, and broader input ranges are areas for future exploration, this work lays the foundation for adaptable, AI-assisted fabrication systems with strong potential in photocatalysis, sensing, and environmental applications.

## Data Availability

All data is available at the request of the corresponding author.
